# An Egalitarian Perspective on Information Sharing: The Example of Health Care Priorities

**DOI:** 10.1007/s10728-023-00475-7

**Published:** 2023-12-30

**Authors:** Jenny Lindberg, Linus Broström, Mats Johansson

**Affiliations:** 1 Department of Clinical Sciences Lund, Medical Ethics, Lund University, BMC I12, Box 117, Lund, 22100 Sweden; 2 Department of Internal Medicine, Skåne University Hospital, Malmö, Sweden

**Keywords:** Informed consent, Shared decision-making, Respect for person, Honesty, Health care priorities

## Abstract

In health care, the provision of pertinent information to patients is not just a moral imperative but also a legal obligation, often articulated through the lens of obtaining informed consent. Codes of medical ethics and many national laws mandate the disclosure of basic information about diagnosis, prognosis, and treatment alternatives. However, within publicly funded health care systems, other kinds of information might also be important to patients, such as insights into the health care priorities that underlie treatment offers made. While conventional perspectives do not take this as an obligatory part of the information to be shared with patients, perhaps through viewing it as clinically “non-actionable,” we advocate for a paradigm shift. Our proposition diverges from the traditional emphasis on actionability. We contend that honoring patients as equal moral agents necessitates, among other principles, a commitment to honesty. Withholding specific categories of information pertinent to patients’ comprehension of their situation is inherently incompatible with this principle. In this article, we advocate for a recalibration of the burden of proof. Rather than requiring special justifications for adding to the standard set of information items, we suggest that physicians should be able to justify excluding relevant facts about the patient’s situation and the underlying considerations shaping health care professionals’ choices. This perspective prioritizes transparency and empowers patients with a comprehensive understanding, aligning with the ethos of respect for the patient as person.

## Introduction

Patients ought to be provided with relevant information about the situation they are in and the options they are facing. Not only is there is widespread agreement that physicians (and sometimes other health care professionals) have a moral duty to provide such information, but in many jurisdictions this is also a legal obligation.^1^ What the justification for information requirements could be is something we shall return to, but for one thing, unless patients are being briefed about their situation and options, their decision to accept or reject a treatment is not appropriately *informed*, and any consent obtained should not be considered valid. In addition, sharing with patients much of what is known could be considered a key aspect of the broader ideals of shared decision-making and person-centered care.

What the scope of the information requirement should be is an important question, as there are arguably limits to the information that can and should be shared with patients. Sharing certain types of information is almost always considered mandatory. These include facts about the patient’s diagnosis and prognosis, and about the available treatment options and their expected effects. Other types of information are rarely if ever listed, however, even though they may be crucial for the patient to fully understand the situation they are in, and the range of options available to them. One example is information about health care priorities, as decisions about how to allocate resources and treatments in a patient population often influence individual treatment decisions. In this paper, we discuss whether there are moral reasons for informing patients about priorities and similar considerations not having to do with what would benefit the individual patient the most. For example, should patients be told that certain diagnostic procedures, observations and potentially beneficial treatments cannot be offered to them because they are too expensive (even though they are likely to accurately confirm or rule out a diagnosis or, in the case of treatment, to be beneficial to them)? And should a patient be told that one reason for not being admitted is that there are not enough hospital beds?

In a publicly funded health care system, rationing is inevitable, and it should not come as a surprise that society has to make some difficult decisions about the allocation of health care resources. Those decisions are not always transparently explained to the public [1], and an interest from citizens [1] and from patients [2] to receive more information about priorities and rationing has been identified. There is scarce empirical data on the extent to which information about priorities is communicated to patients in the individual patient encounter. While patients may have a general awareness of the need for prioritization in health care, they may not know when and how priorities are of direct relevance to their own situation [2]. When they are relevant, in fairly straightforward ways, should this be communicated to patients, even if they do not ask for this information?^2^

The paper is structured as follows. First, we give some examples of when providing prioritization information could be considered in real-world clinical settings. We then turn the attention to policy on patient information (in codes of ethics, regulation, etc.), to determine whether relevant policy frameworks provide a basis for incorporating priorities in what should be communicated to patients. As priorities are currently not explicitly listed, this section of the paper focuses on exploring the *indirect* support found in these normative frameworks for sharing such information. In the subsequent section, we point to recent developments aimed at improving the balance in the physician-patient relationship, including shared decision-making, person-centered care, and health literacy. Such approaches may stress the importance of sharing information with patients, but nonetheless fall short of treating patients as moral equals, we argue, by still assuming a paternalistic view of the purpose of information sharing. On what one may call a more egalitarian view, the perspective on the purpose of sharing information with patients is shifted, as is the burden of proof when it comes to withholding information. On such a view, we contend, letting patients know the extent to which their treatment offers are based on health care priorities and similar considerations, should rather be the default – particularly when patients would otherwise be likely to misunderstand their situation. The final section addresses the many reasons why this alternative perspective should not, however, be seen as supporting an unconditional policy to provide information about prioritization considerations. The article ends with some brief concluding remarks.

## Prioritization Information in the Clinical Setting

In publicly funded health care systems, health care priorities are set at different levels. This article concentrates on priorities that not only have a direct bearing on the clinical offer or recommendation made, but where it may not be obvious to the patient how they are reflected in that offer or recommendation. For the purposes of this paper, we believe there is little need for an exact definition of priorities, or of information about priorities, but here are some examples of the kind of situations we have in mind:

Case 1. Amy arrives at the emergency room, with chest pain and nausea. She is examined, and there is nothing on the EKG or lab results pointing toward acute coronary syndrome (myocardial infarction or unstable angina). The physician thinks that Amy has a reflux problem and prescribes medication for her stomach, and Amy feels a little better. Amy is discharged a few hours later, with the physician telling her that her condition does not warrant her staying at the hospital and advising her to return if her symptoms worsen. Amy is worried and wants to stay in the hospital. From a clinical point of view, admitting her would actually make sense, as it cannot be ruled out that she suffers from more serious conditions, such as angina or peptic ulcer. If there were more hospital beds available, there would be good reasons to keep her overnight for observation and possibly to check more laboratory and vital parameters in the morning. However, with a heavy care load and patients in greater immediate need than Amy seems to be, discharging her makes perfect sense.

Case 2. Bill suffers from chronic kidney disease and is about to start dialysis. The hemodialysis unit at the clinic has limited capacity, so peritoneal dialysis is considered a better option because it is performed at home, and Bill can manage his own dialysis after a period of training. It is also less expensive than hemodialysis. Bill is worried that he will not be able to manage his own dialysis and expresses this to his physician. The physician, however, convinces Bill that peritoneal dialysis is better for him, since it will preserve his residual renal function and be a gentler treatment for him. This is true, but the main reason for choosing peritoneal dialysis is the priority situation.

Case 3. David has an appointment with his general practitioner and complains of a headache of three weeks’ duration. The physician performs a clinical examination which reveals no pathological neurological findings. He suggests that David try some tablets for the headache, reassuring him that it is nothing serious and that there is no need for a CT scan or MRI. Such an investigation could, however, have revealed a brain tumor, which in some cases only presents with headache. In the absence of pathological neurological signs, however, the physician considers a CT scan or an MRI, especially the latter, too expensive.

A common denominator in all these cases is that health care priorities influence the options presented to the patients. This weighing of the interests of one patient against other interests, or against the interests of other patients, impacts the options offered to the individual patient. However, this patient may not understand if and how it does, and may even draw the wrong conclusions about her own health care needs and how they will be met. In contrast, knowing about underlying prioritization considerations would give the patient a more complete picture of the situation she is in, and how the treatment options on offer relate not only to what would best promote her own health the most.

## What do Law and Policy Tell Us?

How should we settle the issue of when, if at all, information about priorities should be communicated to patients? One possible starting point is to look at the rationale that policymakers give for the information requirements they impose. First, there is wide agreement that clinicians (or other health care professionals) do not have total discretion to decide what information to disclose to patients. Rather, patients should be provided with information that is relevant to their situation and the choices they are facing. Since some kinds of information should almost always be seen as relevant, the provision of such information will often be explicitly required. Regarding certain other categories of information, clinicians may have some discretion to decide what to include and what to exclude in any particular case. Even so, however, when assessing relevance, clinicians should be guided by the presumed perspective of patients (actual or “reasonable”), rather than what they themselves consider important.^3^

Across a broad range of influential national and international ethical codes – among them the World Medical Association’s (WMA) International Code of Medical Ethics, and the codes issued by the American Medical Association (AMA) and the United Kingdom General Medical Council (GMC) – there is indeed a common core of information that should be included in conversations with the patient prior to any medical decision-making: basic diagnostic information, what treatments are offered and their expected effects, and the like. However, the moral reasons underlying policies on what information should be included and what information need not be, are often not clearly stated, or they are expressed in such broad terms that they provide little guidance. When, on occasion, a justification for information requirements is given, it typically appeals to overarching goals such as respect for patient autonomy, patient self-determination, and integrity – goals that to varying degrees of depth are frequently also appealed to, or at least considered, in the academic literature [8–13]. It is possible that those ideals could support a policy that patients should be provided with information also about priorities underlying their clinical options. They are often so broad and vague, however, that they typically leave us at square one when trying to settle this matter.

On a more general level, what could be the purpose of information requirements? One purpose could be to ensure that patients’ consent is adequately informed to be legally valid, and to protect clinicians from legal action [10, 11]. If the consent was not properly informed, it would not transfer the legal responsibility to the patient. Relatedly, information can be seen as a necessary condition for patients to be able to exercise their legal right to self-determination in deciding whether to accept the suggested treatment or care, preventing unwanted medical interventions [12]. If meeting the legal threshold for obtaining valid consent was the sole, or main, point of informing patients, informing them about priorities would usually be optional.

Partly related, one could view the main purpose of providing patients with information to be to enable them to act in ways that are in one way or another useful to them. This kind of *actionable* information allows patients to *do* something with it: it might assist them in making risk/benefit assessments, as part of the process of reaching the conclusion whether to accept suggested treatment or care. Or the information could be actionable by facilitating certain other planning. Current guidelines on patient information aim to enable involvement in health care decision-making, allowing patients to choose between treatment options [12]. Now, it is not clear exactly what information policies an actionability criterion would suggest, but again, from that perspective, providing information about the health care priorities underlying the clinical options offered may still be optional, as such information typically will not aid patients in their planning, or make a difference as to what treatment offers one is inclined to accept.

While actionability may be a sufficient condition for valid consent, it is not a necessary condition. Notably, the information currently designated as mandatory for disclosure to patients appears to transcend mere actionability. After all, elements of information such as diagnosis and prognosis may certainly play a crucial role in shaping health care decisions, but they do not have to do so. Patients have the right to be informed about their diagnoses, even in situations where effective treatment options are limited or unavailable. Of course, for almost any type of information, one can imagine situations in which it plays an instrumental part in decision-making. For example, a person who learns about priorities that affect his or her treatment options might be motivated to write an opinion piece in a newspaper. If actionability is to provide real guidance, however, it needs to be limited. The relevant actionability could in this context be framed as *clinical utility*, i.e. information which is useful from a therapeutic or preventive perspective. Borrowing from the field of genetic testing and the return of results to patients, we might say that information considered to be of clinical utility is such that it leads to an “improvement of outcome or the prevention of disease” by allowing for “available proven therapeutic and/or preventive interventions” (p. 579) [14]. Information about priorities needs clearly not be actionable in the narrow or direct sense defined by the concept of clinical utility, as is for example, information about possible treatments and the prevention of future outcomes of a genetic disease.

It appears as if information about priorities does not generally promote rational patient choice or planning *within* a publicly funded health care system, since negotiation is rarely an option (p.163) [15]. But what about treatment provided outside that system? This may, potentially, be something that individual patients would benefit from knowing about, and hence in this sense the information about them can be actionable. Patients can, for instance, turn to private health care providers, whether those providers coexist within the publicly funded system or work through co-payment solutions [16]. Informing patients about such possibilities is mirrored in GMC guidelines, where it is clarified that other information that can be relevant includes “any treatments that you believe have greater potential benefit for the patient than those you or your organization can offer” [17]. Moreover, in Norway [18] and the United Kingdom [19, 20], the question has been raised whether there is an obligation to disclose information about what is *not* covered by the publicly funded health care.^4^ Needless to say, when conveying such information to patients, physicians are arguably required also to explain why that particular treatment is not offered. Therefore, priority considerations arise, and physicians “must use their clinical experience to make specific recommendations while ensuring that the grounds for these recommendations are as transparent as possible” (p. 536) [12].

Focusing too much on actionability could also lead us to unexpected and undesirable moral consequences. If the principle of communicating priority information to patients is based solely on clinical utility, it seems more appropriate to provide this information to the wealthy, who can seek medical advice and treatment anywhere in the world, rather than to, say, less privileged individuals to whom such information might rather be frustrating. This may seem like an unwelcome consequence in publicly funded health care systems that aim to promote equal access to health care, as it would increase inequalities between different socioeconomic groups. Furthermore, it would also be practically challenging to ascertain clinical utility, as that will depend on several factors, of which only some are known to the physician, such as the patient’s prospects of overcoming financial hurdles.

## Widening the Perspective: Shared Decision-making and Respect for Moral Equals

The requirement that the physician should provide the patient with information to enable patient decision-making and meaningful choice between treatment alternatives is a safeguard against outright or “raw” paternalism, but does not take the idea of patient participation particularly far. Current models of shared decision-making, person-centered care, and health literacy have also promoted the idea of sharing important knowledge with the patient, but go further along this path. They, too, assume that patients should be enabled to make free and informed choices between options, and often stress the importance of involving patients throughout the decision-making process [22–29]. While there are several models of shared decision-making [25], the concept revolves around an ideal of shared knowledge and shared responsibility in decisions, involving both knowledge about treatment options and patient preferences, and ultimately the attainment of consensus [22]. In doing so, this ideal of shared decision-making challenges the conventional patient-physician relationship, including traditional models of informed consent, where the physician suggests a treatment option and informs the patient about the medical rationale for choosing that option, and the patient merely consents or dissents. Likewise, person-centered care and the promotion of health literacy view the purpose of information sharing in a partially different way from the way it has traditionally been viewed. Underlying person-centered care are ideals that come down to a holistic perspective of the patient, and partnership in care between the patient and the physician [27, 28], a partnership in which the provision of information could serve broader purposes than merely ensuring the validity of consent, or facilitating actionability, narrowly construed. As for health literacy, it has recently been described as “the degree to which individuals have the ability to find, understand, and use information and services to inform health-related decisions and actions for themselves and others” [29]. This suggests that health literacy, too, aims to empower patient agency by providing patients with more than just the option to consent or dissent. Patients are encouraged to play an active role in prevention, disease management and treatment, to achieve higher levels of health [23, 24, 29].

The relationship between patients and their physicians is asymmetrical. While patients have needs and vulnerabilities that physicians do not share, physicians often possess the ability to address those needs and vulnerabilities in ways the patient cannot. Furthermore, physicians typically have the mandate to determine whether to provide medical assistance and in what manner, whereas patients lack such control. And, of course, there is also a significant difference in what they know about the relevant condition, prognosis, and other relevant circumstances. Some such imbalances may be inevitable – an inextricable part of the patient-physician relationship. But others may not, at least not to the extent typically seen, creating an undesirable situation of asymmetry. The movements toward greater patient participation, mentioned above, could perhaps be understood as efforts to put the parties on more equal terms by increasing patients’ understanding of their condition.

Still, while these more recent approaches highlight a somewhat different role for patients and a correspondingly broader view of the purpose of providing patients with information, they may nonetheless still view information in predominantly *instrumental* terms [22, 27, 29]. And if shared decision-making, for example, is justified by reference to the instrumental benefits of extensive information sharing – that it might, say, ultimately promote the patient’s health or the satisfaction of their preferences, such models will generally still encourage a paternalistic perspective on information sharing, from which providing patients with information about the prioritization considerations underlying offers and recommendations may make little sense. Could there be some alternative (or additional) normative basis for more extensive information sharing requirements, which goes beyond facilitating informed decision-making and choice? Below we suggest one such basis, on which we believe prioritization information could be given a more important role.

Few would deny that human beings are equals in a moral sense, possessing equal moral worth and deserving respect as persons. Why is this relevant in the current context? Respecting someone as a person arguably carries with it certain communicative obligations that go beyond simply conveying actionable information. And if one truly views someone else as an equal, one will regard power imbalances as prima facie problematic, even if some of them do turn out to be unavoidable. Together with this comes, we contend, viewing the possession and sharing of information through an egalitarian lens. The physician’s choosing whether to share with her patients certain information about their situation, based perhaps on whether that information could be expected to be of practical (typically clinical) use to the patients, could be viewed both as an expression of, and as perpetuating, a problematic form of power over her moral equal. It is an expression of power, as it assumes that the physician “owns” the information, gets to decide whether he or she should share it, and allows paternalistic considerations to play a key role in that decision. Moreover, it risks bringing about a power imbalance by making it likely that the patient remains less knowledgeable and, consequently, more vulnerable to another’s continued exercise of power. An egalitarian perspective would rather reverse this, with the normative *starting point* being that health care does not have a stronger right to potentially relevant information than the patient does, or a stronger right to determine relevance. In other words, there is a sense in which the fundamental respect for a person as an equal shifts the burden of proof, so that one would rather need reasons to exclude potentially relevant information from what one shares with patients, than reasons to include it.

Importantly, respecting someone as an equal also comes with a *pro tanto* reason to be *honest* with that person, whether or not the information conveyed benefits the addressee in some more tangible sense, since not doing so also implies wronging the person [30]. What exactly does honesty in this sense involve? A full account obviously cannot be offered here, but honesty certainly involves more than just speaking (what one believes to be) the truth. Because, as we all know, one may be entirely truthful but still provide information which is *misleading*. If one knows this to be the case, or at least should know it, one is not honest in the way one ought to be, being in a respectful relationship. The cases we provided at the outset, the kinds of which we believe there are plenty, illustrate this point. They are cases where the information to a certain extent does truthfully reflect person-centered clinical considerations, but simultaneously conceals other kinds of underlying considerations – ones which patients may not be obviously aware of. The information provided is partly misleading regarding the situation the patients are in and about the norms governing the health care professionals on whose help they must rely.

To see how this might play out in clinical practice, it might be instructive to look at the examples given in Sect. 2. When Amy is told to go home, and only return to the ER if things get worse, she is likely to conclude that there is no significant medical reason for her to stay. However, there is such a reason (by definition) as Amy would be better off staying at the hospital for observation. This reason is just not considered weighty enough, given scarce resources and the more pressing needs of other patients. Similarly, for Bill, what is communicated is his personal benefit of getting peritoneal dialysis, although one primary reason for recommending this relates to priorities. The latter consideration is put on the weighing scale of the physician’s decision-making without the patient knowing about it. It is not a transparent balance, but a disingenuous one in that sense. As with David, the physician takes a risk (on behalf of David) by not performing a CT scan or an MRI. Although the risk of him having a brain tumor is very small, there could be a delay in diagnosis if indeed there was a tumor.

Withholding information regarding personally relevant information can be seen as deception by omission, allowing people to continue to have a false belief, or to be prevented from acquiring a true belief, concerning what is important to them [30, 31], (p. 56) [32]. Providing patients with information that will help them get a more complete picture of their situation, a true belief, can be supported by a basic notion of honesty (p. 257, 265) [32] intrinsic to a relationship of trust (p. 202–203) [32]. Controlling the flow of information, for example by withholding relevant information may be a manifestation of power, making the one who is deceived in some way reduced to not being on equal terms with the one who deceives (p. 19, 282) [33], [34, 35]. Believing that the physician acts for the benefit of the patient while what motivates the decision is also influenced by other factors, betrays the relationship of trust between physician and patient (p. 203) [32]. Allowing, intentionally or just negligently, patients to draw mistaken conclusions about the extent to which offers and recommendations are tailored to their specific situations, and perhaps also about their own health condition, is simply disrespectful and dishonest, we suggest. And especially so, we would argue, when the information concerns such important matters as a person’s health, and the relationship with the professional on whom one must rely in these crucial matters.

It should be stressed at this point that these considerations about honesty and respect obviously do not imply that health care professionals have a *pro tanto* reason to inform patients about everything they happen to know about health care prioritization. Making the physician, for example, responsible for educating his or her patient about national priority models, very remotely connected to what is and isn’t offered or recommended to the patient here and now, makes little sense. Information about overarching priority settings on a national level, about what the health care budget for the next year is, or about certain surgical interventions, unrelated to the patient’s current disease, being centralized to certain hospitals, is indeed information that patients may have to look up elsewhere. The clinical situations under consideration here are, however, importantly different. They not only concern priorities that affect the individual patient in a fairly direct way, but the way in which prioritization considerations influence what options are presented to the patient cannot be deduced from learning about health care priorities in general. Crucially, the situations in which we would argue that physicians may have strong moral reasons to disclose prioritization information are, as already indicated, situations where patients are otherwise at risk of misunderstanding why certain treatments are being offered or recommended, and why some are not; at risk, in particular, of drawing the wrong conclusions about their own health condition.

Now, one might worry that being frank about rationing or prioritization considerations would adversely affect patients’ inclination to have *trust* in their physicians (or other health care professionals with the responsibility to provide relevant information), since transparency regarding health care priorities and similar considerations will make it obvious that the physician is not only guided in medical decision-making by the patient’s personal benefit from a treatment, but also considers other important factors. Any image of the physician as merely the patient’s advocate will inevitably vanish. Not only is this conjecture quite speculative, as hypotheses about something so obviously complex as how various factors contribute to, or detract from, trusting relationships would have to be backed up by evidence. More importantly, however, it is irrelevant. Because even under the assumption that trust would indeed be negatively affected if physicians were more forthright about prioritization considerations, a case could certainly be made that such an empirical finding would have little to do with what the respectful thing to do is. As a matter of fact, if physicians were to be trusted more by not being transparent about the reasons underlying their offers and recommendations, that trust would arguably be *undeserved*. Furthermore, patients are exposed to, and actively have to navigate between, various sources of information. Consequently, they may themselves find out that treatment options are being withheld without knowing the reason for it, and that could, in turn, harm the physician-patient relationship [36]. This also points to a different reason why a respectful approach towards equals calls for transparency in this context. Not only do general norms about honesty suggest that the relevant information should be conveyed (unless there are specific reasons to the contrary). It could also be argued that it is quite obviously disrespectful to *diminish* individuals by simply assuming that they would not be able to handle the fact that concerns about others, demanding prioritization, also are taken into account in health care decisions.

## Now What? The Old Problem in a New Outfit?

We have suggested that the burden of proof ought to be shifted when it comes to the question of what information should be conveyed to the patient. Choosing *not* to address the issue of prioritization is, on this account, what needs justification. However, in clinical practice there will indeed be good reasons, of various kinds, not to share certain information with the patient. This means that no concrete policy follows just from rethinking information obligations along the lines we have suggested. For one thing, in any real-world scenario time will be limited, and it will for that reason alone be impossible for physicians to convey everything that is conceivably relevant. Sometimes circumstances will dictate that the physician must be particularly brief. In cases where other patients’ lives and well-being are at stake, for instance, it will be morally acceptable to interrupt a conversation with a patient, or to reduce the time allocated to it, concentrating the information to the minimum required for informed consent or dissent. This means one needs to prioritize among all the things that could be communicated to the patient.

Of course, it is not only lack of time that calls for such information priorities. Most importantly, information should be *understood* by the patient in order not to be misleading. Trying to share “all of it” may be in conflict with that goal. When not only complex medical knowledge is being communicated to patients, but also other information, there will often be a legitimate concern about causing information overload, which rather risks eroding patients’ understanding of treatment options by making them more confused, creating false rather than true beliefs. The patient may simply be unable to process all the information, or be distracted by the sheer amount of it, so that the most important aspects of it are lost.

Potentially, information can also cause harm – for example, by making the patient seriously worried, sad, or ashamed, or by frustrating hope and abilities. Such emotional reactions might be especially challenging for some vulnerable patients, and the risk of harm might outweigh whatever interest the patient nevertheless has in receiving the information. It might also be better not to convey information that, because of this emotional response, might lead to misconceptions instead of true beliefs in the patient. We have no doubt that prioritization information could cause these reactions, depending on the particulars of the patient and the situation at hand. (On the other hand, we see no reason to believe that it will typically do so more than other kinds of information.)

For the reasons just given, and others, it does not follow from the egalitarian view on information sharing sketched, that in every situation, patients ought to be given information about priorities. There may indeed be situations in which the information is best withheld. As with other kinds of information, one may have to adapt it to the needs of each individual patient. The point, again, is just that, from this perspective, the presumption is that information, if it contributes to conveying a truthful picture of the patient’s condition and possibilities, should be shared unless there are convincing considerations to the contrary. The onus is on the health care professional to be able to explain why prioritization information could, or even should, be left out of what is shared with the patient.

Also, nothing in the egalitarian perspective suggested here precludes giving special weight to what is particularly *useful* to the patient, in the situation at hand. As previously mentioned, utility and actionability can be assessed in various ways. But when physicians need to be selective with respect to what they share with patients it certainly makes sense to give greater weight to information that can be expected to affect the patient’s deliberate decision-making and preparation for what lies ahead. Adjustments may need to be made by the patient to his or her work life, family life, or plans in other respects that could be affected by disease, rehabilitation, disability, etc. Information sharing could, moreover, have more indirect instrumental value, as it does, for example, when it serves to promote trust in health care, which in turn facilitates future interactions with health care professionals.

Depending on the situation, prioritization information may be crucial from an instrumental perspective. But again, non-instrumentalist considerations may apply as well. For example, to show basic respect for patients one must arguably try to make them understand *why* they are being offered (or recommended) the treatment they are. If no mention is made by the physician of the reasons for prioritization, patients may easily be misled and believe that clinical superiority is the (only) reason they are being given the relevant option. And knowingly allowing patients to develop such false beliefs could certainly be considered inconsistent with the ideal of respecting others as equals. The importance of information, in this case, is rather connected with norms implying that one must not intentionally mislead a person of equal moral standing because this is a form of epistemic injustice [35].

More generally, respect for the patient as person could be shown by sharing information that clarifies roles and relationships, including power relationships, and helps the patient recognize their own position in a community of equals with equal claims. Sharing information about priorities can also help the patient to better understand a personal choice. For example, it could involve recognition of the possibility that the patient may wish to altruistically sacrifice some of his or her health or safety for the good of others. Regardless of whether the information proves useful to the patient this would mean that the physician is taking the patient’s role, not only as a stakeholder, but also as a moral subject, seriously.

To sum up, sharing information about priorities needs to be balanced against other values or interests, including competing information needs given the time allocated, the risk of contributing to misunderstandings or that the information may cause harm. Ideally, the information should be tailored to the individual patient´s needs and preferences, because not all patients want or need the same amount of information [37]. On the other hand, general standards and guidelines will be required. To reiterate, we are not here proposing to modify policies or provide detailed guidelines. We do, however, want to highlight what we consider to be a paradigm shift in the conception of information sharing. When considering new guidelines, we suggest they reflect the egalitarian shift outlined here, matched with honesty regarding personally relevant information as a form of respect. Priority considerations, when relevant to understanding treatment options, should arguably be conveyed by default. Any exclusion of this information would need to be justified from an egalitarian perspective.

The egalitarian approach may seem to open the floodgates to information sharing. However, it should better be seen as encouraging us to rethink the ethical reasons for withholding information in the first place. Information that concerns the patient is not limited to what is useful for the patient, but what this means in terms of modified guidelines and/or clinical practice is still open to discussion. More work is needed to settle on the practical implications of the egalitarian approach, which takes into account many of the practical circumstances surrounding modern health care.

## Footnotes

1 Such is the case in a wide range of national legislation, e.g. in the United States (with variations according to state), Australia (Australian Charter of Health Care Rights), the United Kingdom in its common law preventing battery, Canada, and Sweden (the Swedish Patient Act).

2 In health care systems where all or a part of the cost is paid by patients, there are data suggesting that the public wants information about treatments that may be given at a greater expense, if these treatments contribute to prolonged survival, quality of life, or if there is no other treatment available [3]. For the complexities of discussing financial issues with patients in health care systems where the patient pays for a major part of the cost of care, such as the United States, see also e.g. [4].

3 A *reasonable patient* standard [5] should not be confused with the far more demanding *subjective standard* – a standard which few jurisdictions have adopted and which “judges the adequacy of information by reference to the specific informational needs of the individual person rather than by the hypothetical reasonable person” (p. 125) [6]. Building on what is reasonable to someone is obviously not limited to clinical settings, but has for example also been discussed in the context of research [7].

4 This has been considered the case in a Swedish legal context (p. 214–215) [21].
